# Identification of Proteins Responsible for the Neuroprotective Effect of the Secretome Derived from Blood Cells of Remote Ischaemic Conditioned Rats

**DOI:** 10.3390/biom12101423

**Published:** 2022-10-04

**Authors:** Petra Bonova, Jana Koncekova, Miroslava Nemethova, Klaudia Petrova, Martin Bona, Miroslav Gottlieb

**Affiliations:** 1 Institute of Neurobiology, Biomedical Research Center of the Slovak Academy of Sciences, Soltesovej 4-6, 040 01 Košice, Slovakia; 2 Department of Medical Physiology, Faculty of Medicine, University of Pavol Jozef Safarik, 040 01 Košice, Slovakia

**Keywords:** blood cell, secretome, protein, bioactivity, neurotoxicity, protection, haptoglobin

## Abstract

We have recently shown that the blood cell-derived secretome of remote ischaemic (RIC)-conditioned individuals provides an external source of neuroprotection. In this study, we identified the bioactive compounds from the total proteins released by those cells. Our main strategy was to separate protein–protein complexes while maintaining their native structure and testing their bioactive properties. Subsequently, we identified up- and downregulated bioactive proteins. We uncovered two bioactive fractions composed of 18 proteins. Most of the protein peaks were unchanged; however, RIC mediated a decrease in two peaks (comprising seven proteins) and an increase in one peak (identified as haptoglobin). When focussing on the biological activity of these proteins, we found positive impacts on the regulation of cellular metabolic processes and an increase in biological processes related to the acute phase response and inflammation in the RIC-treated samples. Although we have identified the 18 proteins that exert the greatest cytoprotection, additional studies are needed to elucidate their particular function and detailed mechanisms of action.

## 1. Introduction

Stroke is the second leading cause of death and disability worldwide. There are two types: haemorrhagic stroke, the most common, and ischaemic stroke. Currently, there is only one Food and Drug Administration (FDA)-approved treatment for ischaemic stroke in clinical practice, namely the application of tissue thrombotic plasminogen activator (tPA) antithrombotic agent. However, oftentimes tPA cannot be administered because it has been too long since the stroke occurred (<4.5 h) and there is frequent haemorrhagic turnover [1].

Recently, remote ischaemic conditioning (RIC) has been used more frequently in clinical practice to alleviate the impacts of stroke. The basis of RIC is endogenous stimulation of neuroprotective mechanisms by using distant organ or limb ischaemia. Blood flow occlusion to a limb via application of external pressure initiates transmission of a signal that mediates the RIC response. Humoral, neurogenic, and immune-mediated pathways have been proposed in the transmission and end-organ effector mechanisms. After transmission of the conditioning stimulus, a myriad of possible biochemical and physiological changes may contribute to clinically meaningful outcomes in stroke patients. The positive neuroprotective effect of RIC has been reported to correlate with improved brain blood flow, angiogenesis, and collateral blood supply; haemostasis and an antiplatelet effect; immunoregulation; and synaptic plasticity (reviewed in [2]).

On the other hand, some studies have reported the inefficiency of RIC in different disease settings. Researchers suggest that older age and the female sex suppress RIC-mediated protection. Similar to those non-modifiable risk factors, RIC-mediated protection is abrogated in individuals with comorbidities, such as hypercholesterolaemia, diabetes, and hypertension, probably because a low RIC dose was applied. The dosing could be considered a disadvantage of RIC in other patients. In most of the published studies, one dose of RIC has consisted of 3–5 cycles of limb ischaemia, each lasting 5–10 min. The dose is applied several times a day for 2–5 days, an approach that may cause neurovascular damage and tissue lesions in some individuals [3]. Therefore, omission of direct limb ischaemia as part of the RIC protocol could represent a significant improvement in the treatment of some special cases. The solution for these patients who are intolerant to RIC may be utilising the blood cell-derived secretome of healthy RIC-treated individuals.

There is ample evidence that RIC-mediated neuroprotection could be transferred indirectly via blood transfusion [4,5] or the blood cell-derived secretome drawn from RIC-stimulated animals [6]. In our recent work, we discovered that protein synthesis is crucial for the induction of tolerant phenotype in ischaemia-affected animals. The blood cell-derived secretome of RIC-stimulated animals comprises both commonly and uniquely secreted proteins involved in the regulation of biological processes, cellular processes, and responses to stimuli and chemicals. However, the components responsible for the induction of neuroprotection against ischaemia have not been identified.

In this study, we identified the bioactive proteins in the blood cell-derived secretome of RIC-stimulated animals. Because the natural structure is essential for the bioactive properties of each protein and the clustering of proteins into bioactive complexes should not be excluded, we chose to separate the blood-derived protein mixture under native conditions. We also determined the protein fractions with a neuroprotective effect and their role in particular biological processes.

## 2. Materials and Methods

### 2.1. Ethical Statement

The experiments were performed in accordance with an animal care protocol that was approved by the European Communities Council Directive (2010/63/EU) and with permission from the State Veterinary and Food Administration of the Slovak Republic (4451/14-221 and 4247/15-221) under the supervision of the Ethical Council of the Institute of Neurobiology BMC SAS. We made every effort to reduce the number of animals used and to minimise animal suffering. We maintained adult male albino Wistar rats (bred at the certified vivarium of the Institute of Neurobiology BMC SAS and originating from Velaz Ltd., Praha, Czech Republic) weighing 100–120 g (approximately one-month old, *n* = 6) or 270–320 g (approximately 3 months old, *n* = 20) on a 12 h photoperiod and given food and water ad libitum.

### 2.2. Experimental Design

**Figure 1:**
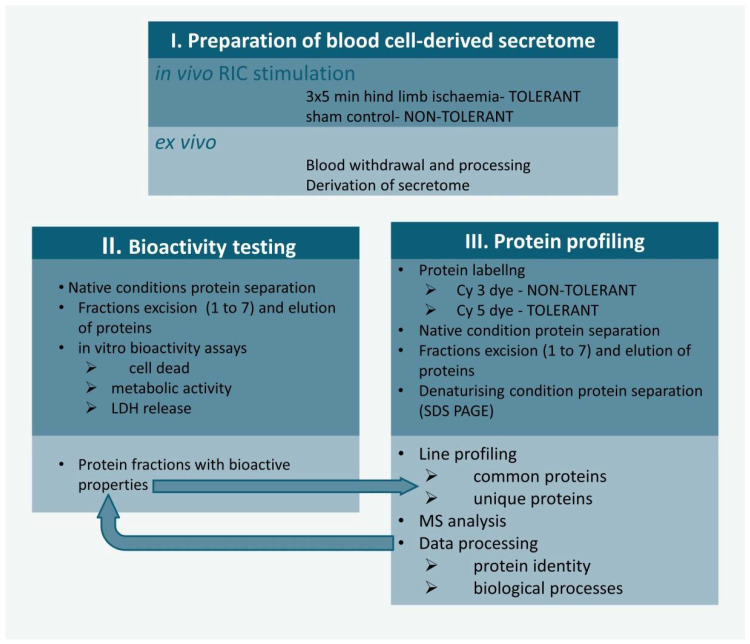
Experimental design. (**I**) RIC stimulation/non-stimulation of rats via limb ischaemia followed by the preparation of blood cell-derived secretomes. (**II**) Protein mixtures were separated with native polyacrylamide gel electrophoresis (PAGE) and partitioned into seven fractions. Proteins were passively eluted from the gel pieces, and the bioactivity of proteins was tested for each fraction. (**III**) Cy3- and Cy5-labelled proteins were separated into the tolerant/non-tolerant secretome with native PAGE, and then partitioned into seven fractions. Proteins were passively eluted from gel pieces. Proteins were subjected to semiquantitative analysis of uniquely secreted proteins and/or changes in common protein contents, followed by mass spectrometric (MS) analysis of proteins.

Figure 1 provides an overview of the experiments. (I) RIC stimulation/non-stimulation of rats via limb ischaemia was followed by the preparation of blood cell-derived secretomes drawn from RIC-treated (tolerant secretome) and non-treated animals (non-tolerant secretome). (II) Protein mixtures were separated with native polyacrylamide gel electrophoresis (PAGE) and partitioned into seven fractions. Proteins were passively eluted from the gel pieces, and the bioactivity of proteins was tested for each fraction. (III) Cy3- and Cy5-labelled proteins were separated into the tolerant/non-tolerant secretome with native PAGE and then partitioned into seven fractions. Proteins were passively eluted from gel pieces. Proteins were subjected to semiquantitative analysis of uniquely secreted proteins and/or changes in common protein contents, followed by mass spectrometric (MS) analysis of proteins related to bioactive fractions.

RIC-remote ischaemic conditioning; LDH-assaying of extracellular lactate dehydrogenase; Cy 3 and Cy5 dyes- cyanine dyes; SDS PAGE sodium dodecyl sulphate-polyacrylamide gel electrophoresis; MS-mass spectrometric analysis of proteins.

### 2.3. Preparation of the Blood Cell-Derived Secretome

In vivo stimulation via rapid RIC was employed to induce the paracrine activity of blood cells to promote a tolerant phenotype [7]. Right hind-limb ischaemia was induced by placing an elastic rubber band tourniquet on the proximal part of the limb for 5 min, followed by a 5 min period of reperfusion. The procedure was performed under light isoflurane anaesthesia (0.5%) in three cycles, 1 h before blood sampling. Non-tolerant animals underwent the same procedure without the tourniquet application. Heparin (Zentiva International a.s.) was injected intraperitoneally into the donor animal at the same time (250 U/kg). The blood of the RIC-stimulated (tolerant) rats and non-stimulated (non-tolerant) rats was collected by cardiac punction. The blood plasma was discarded after centrifugation (4500× *g*, 15 min, 4 °C). The pellet, which represented a mix of all blood cell types, was washed and resuspended in artificial plasma (hydroxyethyl starch [HES] 130/0.4 in an isotonic electrolyte solution, 6% Volulyte, Fresenius Kabi Deutschland GmbH) to maintain the original blood volume. Subsequently, the samples were incubated in a CO_2_ incubator (37 °C for 150 min) followed by centrifugation (4500× *g*, 15 min, 4 °C). The supernatant was removed and spun again (15,000× *g*, 15 min, 4 °C) to discard the residual cells and cellular debris. The final supernatant represented the blood cell-derived secretome with preserved exosomes and microparticles. The relative protein concentration of the secretomes was 0.6–0.72 mg/mL; it was assessed according to a previous study [8]. The secretome samples were used (1) for in vitro assays or (2) to determine protein differences between the tolerant and non-tolerant secretomes.

### 2.4. Bioactivity Testing

Proteins in secretome samples were separated with native PAGE to maintain the composition and structure of native proteins. The tolerant and non-tolerant secretomes were processed separately for in vitro assays or were pooled after the Cy3 or Cy5 labelling to determine differentially expressed proteins and for subsequent mass spectrometric profiling.

#### 2.4.1. Native PAGE

Proteins in the tolerant and non-tolerant secretomes were separated with native PAGE to maintain the secondary structure of proteins and the native charges. Briefly, the samples were mixed with the sample buffer (62.5 mM Tris HCl, pH 6.8; 25% glycerol; 1% Bromphenol Blue) 1:1 (*v*/*v*) and resolved by 10% native PAGE on ice for approximately 75 min (at 150 V). The bands were cut into 1 cm pieces (seven pieces) and washed in phosphate-buffered saline (PBS). The pieces were placed in Eppendorf tubes containing 1 mL of Neurobasal A medium (Gibco, Thermo Fisher Scientific, Waltham, MA, USA) and proteins were allowed to passively elute out of the gel slices for 24 h at 4 °C. The eluates were concentrated using the Vivaspin ultrafiltration spin columns with a 3000 kDa molecular weight cut-off (Vivaspin 500, Sartorius, Göttingen, Germany).

#### 2.4.2. In Vitro Assays

##### Cell Culture

The secretomes were tested in vitro in primary cultures of adult rat cortical neurons. Cortical neurons were isolated from the brains of 1-month-old male rats, according to previously described methods [9]. Swiftly, rats were decapitated and their brains were removed. The cortical lobes were dissected and sliced at 0.5 mm, digested with papain, and triturated in Hibernate-A/B27 medium (Gibco). The cells were then separated from the debris on a density gradient of OptiPrep^TM^ (Sigma-Aldrich, Saint-Louis, MI, USA). The neuron-enriched fraction was collected, and the viable cells were counted via the trypan blue exclusion test. The cells were plated at 350 cells/mm^2^ on polylysine-coated coverslips (50 µg/mL in water) and cultured in Neurobasal A/B27 medium (Gibco) with 5 ng/mL FGF2 (Invitrogen, Waltham, MA, USA) for 12–15 days (37 °C, 5% CO_2_ and 9% O_2_ under saturated humidity). MAP2 immunopositivity was tested at 10 days in vitro (DIV10) for each cultivation. After fixation in 10% formaldehyde (10 min, room temperature, Sigma Aldrich, Burlington, MA, USA), the cells were permeabilised, and the nonspecific sites were blocked with 3% normal horse serum (30 min, room temperature). Rabbit anti-MAP2 (diluted 1:100; Sigma-Aldrich) was applied overnight at 4 °C. After rinsing in 0.1 M PBS with 0.3% Triton X-100, secondary goat anti-rabbit IgG conjugated to Alexa Fluor 488 (diluted 1:2000; Molecular Probes, Eugene, OR, USA) and 4′,6-diamidino-2-phenylindole (DAPI; Thermo Fisher Scientific) were applied at room temperature for 1 h. The mounted slides were photographed with an Olympus BX51 microscope with a camera (Olympus DP50). The number of MAP2-positive cells was counted and compared with the total numbers of DAPI-labelled nuclei. Only the cell cultures with MAP2 immunopositivity > 75% were included in the analysis.

##### Cell Toxicity and Viability Assays

To test the bioactivity of the protein fractions derived from the native secretomes, the cortical neurons were incubated for 1 day in FGF2-free Neurobasal A/B28 media with 200 µM glutamate (Sigma-Aldrich) and the blood cell secretome (non-tolerant/tolerant, 1:1 *v*/*v*). Each sample was examined in four replicates of three cultivations.

Cell viability was measured with the lactate dehydrogenase (LDH) and 3-(4,5-dimethylthiazol-2-yl)-2,5-diphenyltetrazolium bromide (MTT) assays. LDH activity after the treatment was assessed fluorimetrically in the cultivation media, based on the NADH resulting from the reaction of lactate and NAD^+^ catalysed by LDH. After centrifugation (12,000× *g*, 10 min, room temperature), medium samples were incubated in reaction buffer (0.2 M TRIS, pH 8.6), containing 1.5 mM NAD^+^ (Sigma-Aldrich) and 10 mM lactate (Sigma-Aldrich) in black 96-well plates. After incubation for 30 min at 37 °C, the fluorescence intensity of the final product (NADH) was detected on a Synergy™ 2 Multi-Mode Microplate Reader (BioTek) at 460 nm (with an excitation wavelength of 360 nm). The LDH activity was calculated from a standard curve based on rabbit muscle LDH (Sigma-Aldrich) in the range of 0–2500 U and is expressed as a percentage of the non-treated neurons (i.e., the cell culture exposed to 200 µM glutamate without the addition of secretomes).

The metabolic activity of the cells was determined using a Vybrant^®^ MTT Cell Proliferation Assay kit (Molecular Probes), according to the manufacturer’s instructions. Briefly, the cultivation medium was replaced with a no-phenol red alternative and 10 µL of 12 mM MTT in PBS was added; the plate was incubated for 4 h at 37 °C. Then, the medium was removed, and 50 µL of dimethyl sulphoxide (DMSO) was added to lyse the cells. The absorbance was measured 10 min later at 540 nm. The metabolic activities of the cells are expressed as a percentage of non-treated neurons (i.e., the cell culture exposed to 200 µM glutamate without the addition of secretome samples).

##### Cell Death Detection

Propidium iodide (PI, 1 µM, Sigma-Aldrich) and Hoechst 33258 (1 µM, Sigma-Aldrich) were added to the medium and the cells were incubated for 30 min. The number of PI-positive cells was counted by a blind investigator under an inverted fluorescence microscope (Nikon ECLIPSE Ti) and compared with the total numbers of Hoechst-labelled nuclei. The results are expressed as the percentage of PI-positive cells of the total cell counts.

#### 2.4.3. Proteomic Analysis of Blood Cell-Derived Secretomes

##### Labelling Proteins with Fluorescent Dyes

The pH of the secretome samples was adjusted to 8.6 using bicarbonate buffer (pH 10.1). The protein content in 250 µL of tolerant and non-tolerant secretome samples was randomly labelled using 0.5 µL of Cy3 and Cy5 fluorescent dyes (Lumiprobe, 0.5 pmol/µL in *dimethylformamide*) and incubated overnight at 4 °C. Cy3- and Cy5-labelled samples were pooled (Cy3 + 5) and the volume of the mixture was reduced up to 70 µL by using Vivaspin ultrafiltration spin columns with a 3000 kDa molecular weight cut-off. After that, the samples were subjected to native PAGE, as described in Section 2.4.1. Separated Cy3 + 5 labelled proteins were visualised in gel via a fluorescence laser scanner (Typhoon Trio, GE Healthcare). Then, the lanes were cut into 1 cm pieces, and proteins were allowed to passively elute into 1 mL of PBS for 24 h at 4 °C. After that, eluates were concentrated using Vivaspin ultrafiltration spin columns with a 3000 kDa molecular weight cut-off. Eluted Cy3 + 5-labelled proteins were stored at −80 °C for subsequent separation of non-reduced protein complexes under reducing conditions, namely sodium dodecyl sulphate (SDS)-PAGE.

##### SDS-PAGE and Sample Processing for Mass Spectrometric Analysis

Proteins eluted from gel fractions were separated with SDS-PAGE. Briefly, protein samples derived from native gel fractions were mixed with Laemmli sample buffer and heated at 95 °C for 5 min. After that, the samples of the fractions were loaded in numerical order (from 1 to 7) in 12% SDS-PAGE gels and run at 200 V for approximately 90 min in duplicate. The separated proteins were visualised in the gel via a fluorescence laser scanner (Typhoon Trio, GE Healthcare, Chicago, IL, USA) and analysed using the ImageQuant TL software (GE Healthcare) to profile the lane of interest and to detect RIC-mediated changes in protein bands (area of the peak as mm^2^ and band percentage of the total area of the peaks in lane as a band %).

Lanes 3 and 4, corresponding to fractions 3 and 4, respectively, had the highest bioactivity based on the results of in vitro assays and were processed for subsequent MALDI TOF/TOF protein profiling. Briefly, each lane was cut into 12 pieces (0.5 cm each), minced into small squares, and washed in distilled water. Then, the gel pieces were shrunk by two rounds of acetonitrile wash (15 min, room temperature) and processed for MALDI analysis as described below.

##### Mass Spectrometric Analysis and Data Processing

The protein profiles of the non-tolerant and tolerant secretomes were analysed using MALDI TOF/TOF mass spectrometry (matrix-assisted laser desorption/ionization time-of-flight/time-of-flight). The proteins of gel pieces were digested overnight in a solution with bovine trypsin (Roche Diagnostics, Bazilej, Switzerland) in ammonium bicarbonate buffer (a 1:50 enzyme-to-protein ratio) at 37 °C. The resulting peptides were purified using ZipTip C18 pipette tips (Millipore, Burlington, NJ, USA) and injected into the MALDI target (AnchorChip™ 384, Bruker Daltonics, Bremen, Germany) with an α-cyano-4-hydroxycinnamic acid MALDI matrix (HCCA; Bruker Daltonics). The peptide spectra across the range of 700 to 3500 Da were acquired with the MALDI TOF mass spectrometer (UltrafleXtreme Bruker Daltonics) in positive reflectron mode and analysed with the Mascot MS/MS ions search engine (Matrix Science, London, UK) against the Swiss-Prot database with the tolerance set to 100 ppm for the MS and 0.5 Da for the MS/MS data. The ProteinExtractor algorithm was applied to filter the non-redundant proteins. A 95% confidence interval threshold (*p* < 0.05) was set to identify the proteins, and a positive identification was based on at least one unique peptide and a false discovery rate of less than 1% (FDR < 1%). The web-based Search Tool for the Retrieval of Interacting Genes/Proteins (STRING) database version 11 (http://string-db.org, accessed on 3 June 2022) [10] was used to analyse the interacting proteins with the minimum required interaction score set to 0.7 (high confidence). The main goals of this search were to identify the Reactome pathway of proteins and integration within biological processes.

### 2.5. Data Analysis and Statistics

The data are expressed as the mean ± the standard deviation (SD). We did not perform an outlier test on the data. We used one-way analysis of variance (ANOVA) followed by Tukey’s multiple comparisons test and Student’s *t*-tests or two-way analysis of variance (ANOVA) followed by Sidak’s multiple comparison test. The normality of the data was assessed by D’Agostino–Pearson omnibus test (GraphPad Prism 6.01). The coefficient of variation was calculated as a part of the column statistic (GraphPad Prism 6.01).The false discovery rate (FDR), which describes how significant the enrichment is, is shown as a *p*-value corrected for multiple testing within each category using the Benjamini–Hochberg procedure.

## 3. Results

### 3.1. Neuroprotective Effects of Blood Cell-Derived Secretomes

We divided the blood cell-derived secretomes of the tolerant and non-tolerant animals into seven fractions, and then separated the proteins with native PAGE. Therefore, each fraction consists of proteins in their native structure and unchanged composition of protein complexes, and thus with minimal influence on protein bioactivity. We subsequently tested the fractions in vitro for their protective features on neurons exposed to a toxic dose of glutamate. We selected the gel fractions that showed the highest neuroprotective effect to examine the protein profiles. Our main criteria to select the bioactive factions were metabolic activity, LDH release, and cell death.

#### 3.1.1. Proteins of Fractions 3 and 4 Reduce Neuronal Death

**Figure 2:**
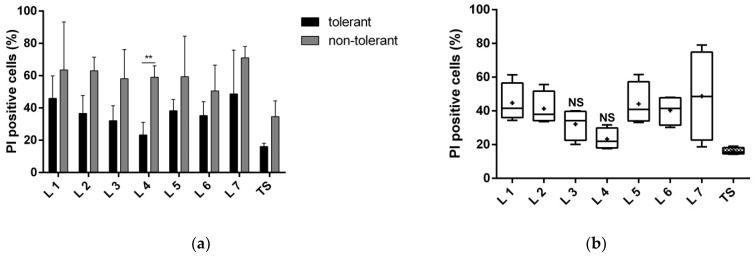
The number of dead cells exposed to 200 µM glutamate and co-cultivated with the total tolerant or non-tolerant secretomes and protein fractions of the total secretomes. Protein fractions (1–7) were separated with native polyacrylamide gel electrophoresis. The number of PI-positive cells is expressed as a percentage of the total number of cells. The tests were performed after 24 h of incubation in four replicates of three cultivations. (**a**) Comparison of the neuroprotective effect of the tolerant versus non-tolerant secretomes and their respective fractions. The data are presented as the mean ± standard deviation (** *p* < 0.01). (**b**) Comparison of the neuroprotective effects of the individual fractions of the tolerant secretome to the total secretome. The graph presents the minimum to the maximum values with the median bar; + indicates the mean. NS, nonsignificant changes in the dead cell count, compared with the total secretome; TS, total secretome.

Our results showed that the positive effect of tolerant secretome on neuronal survival after exposure to glutamate could be mediated by the proteins in fractions 3 and 4. Co-cultivation of the neurons with the protein content of each lane improved survival. However, only fraction 4 significantly reduced the number of PI-positive dead cells, by 37% compared with fraction 4 of the non-tolerant secretome (Figure 2a). When we compared the protective effect of fraction 3 with the total secretome, there was a comparable number of dead cells (Figure 2b).

#### 3.1.2. Proteins of Fractions 3 and 4 Reduce the Toxic Effect of Glutamate and Improve Neuronal Viability

**Figure 3:**
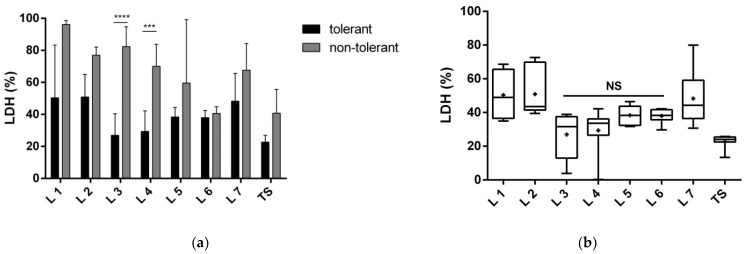
Lactate dehydrogenase (LDH) released by neurons after exposure to 200 µM glutamate and co-cultivation with the total tolerant or non-tolerant secretomes or their fractions. Protein fractions 1–7 of the total secretomes were separated with native polyacrylamide gel electrophoresis. The LDH content is expressed as a percentage of neurons exposed to 200 µM glutamate. The tests were performed after 24 h of incubation in four replicates of three cultivations. (**a**) Comparison of the neuroprotective effect of tolerant versus non-tolerant secretomes and their respective fractions. The data are presented as the mean ± standard deviation (*** *p* < 0.001; **** *p* <0.0001). (**b**) Comparison of the neuroprotective effect of the fractions of the tolerant secretome to the total secretome. The graph shows the minimum to the maximum values with the median bar; + indicates the mean. NS, nonsignificant changes in the LDH content, compared with the total secretome; TS, total secretome.

Compared with the non-tolerant secretome, neurons subjected to glutamate-mediated damage and treated with any fraction of the tolerant secretome showed decreased LDH release. The proteins of fractions 3, 4, 5, and 6 exerted the greatest cytoprotective effects and did not differ significantly from the effect of the total secretome (i.e., those fractions provided the same level of neuroprotection; Figure 3b). However, only fractions 3 and 4 produced significantly higher neuroprotection, compared with their non-tolerant counterparts (about 55% for fraction 3 and 40% for fraction 4; Figure 3a).

**Figure 4:**
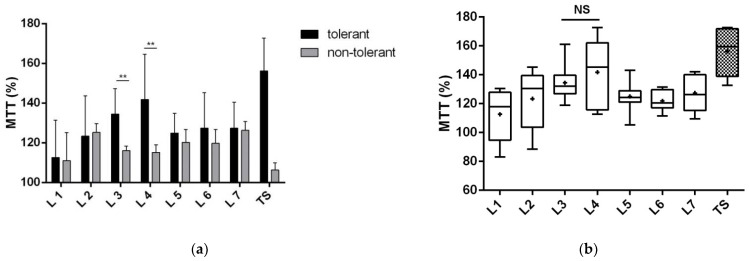
The metabolic activity of primary cortical neurons co-incubated with 200 µM glutamate and the total tolerant or non-tolerant secretome or its fractions. Protein fractions 1–7 of the total secretomes were separated with native polyacrylamide gel electrophoresis. The 3-(4,5-dimethylthiazol-2-yl)-2,5-diphenyltetrazolium bromide (MTT) concentration is expressed as a percentage of non-treated neurons exposed to 200 µM glutamate. The tests were performed after 24 h of incubation in four replicates of three cultivations. (**a**) Comparison of the neuroprotective effect of tolerant versus non-tolerant secretomes and their respective fractions. The data are presented as the mean ± standard deviation (** *p* < 0.01). (**b**) Comparison of the neuroprotective effect of the fractions of the tolerant secretome to the total secretome. The graph shows the min to max with the median bar; + indicates the mean. NS, nonsignificant changes in dead cell count, compared with the total secretome; TS, tolerant secretome.

The metabolic activity of neurons exposed to glutamate was also increased by 156.1 ± 6.8% after co-incubation with the tolerant secretome (Figure 4a). Fractions 3 and 4 produced a similar effect to the total secretome, with a 134 ± 4.5% and 142 ± 7.6% increase in metabolic activity, respectively (Figure 4b). Those fractions showed significantly higher metabolic activity than the respective fractions of the non-tolerant secretome (by about 19% and 27%, respectively).

### 3.2. RIC-Mediated Changes in Proteins of the Blood Cell-Derived Secretome

We incubated the blood cells of RIC-treated and non-treated animals ex vivo to compare protein changes triggered by RIC. We labelled the proteins of tolerant and non-tolerant secretomes with two variants of Cy fluorescent dyes (Cy3 and Cy5)—each with different excitation and emission spectra—then mixed the samples and separated them into seven fractions with native PAGE. We then denatured and separated the proteins with SDS-PAGE and examined the profiles of each lane. Based on the density of the bands, we calculated the peak area to identify unique protein bands/peaks and to calculate semi-quantitatively changes in common peaks. Our main goal was to document the profiles of lanes 3 and 4 (fractions 3 and 4, respectively). We then profiled lanes 3 and 4 with MALDI TOF/TOF. We excised nine pieces (0.5 cm) from each lane and individually analysed them to identify proteins. Based on the peak layouts, we divided the lane profile into six parts. We calculated the peak area (mm^2^) and its percentage of the total area of the peaks in the lane (band %), and we defined the locations on the lane profile (mm from the top of the lane) (Supplementary Figure S1).

**Figure 5:**
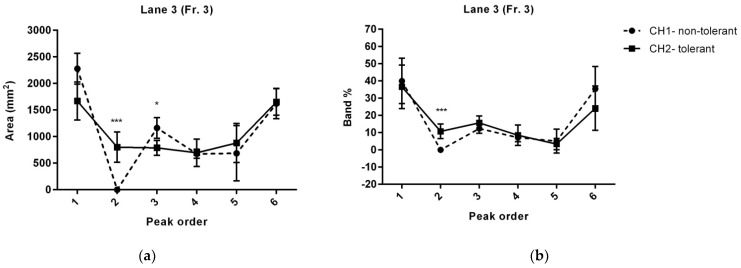

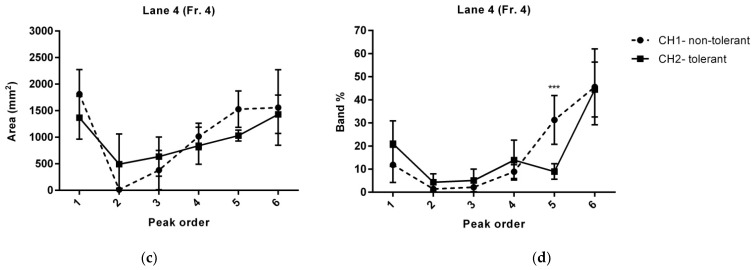
Lane profiles of protein peaks from fractions 3 and 4 after separation with sodium dodecyl sulphate–polyacrylamide gel electrophoresis. (**a**) Peak area (mm^2^) and (**b**) percentage of the total area of the peaks in the lane (band %) of proteins derived from fractions 3 of tolerant (CH1) and non-tolerant (CH2) secretomes. (**c**) Peak area (mm^2^) and (**d**) percentage of the total area of the peaks in the lane (band %) of proteins derived from fractions 4 of tolerant (CH1) and non-tolerant (CH2) secretomes. The data are presented as the mean ± standard deviation (* *p* < 0.05; *** *p* <0.001). Fr., protein fraction separated by native polyacrylamide gel electrophoresis; CH, fluorescence channel of tolerant/non-tolerant proteins.

We have summarised the common proteins in Table 1 and Figure 5. The majority of the peaks—namely peaks 1, 4, 5, and 6 of lane 3, and peaks 1, 2, 3, 4, and 6 of lane 4—represent commonly secreted proteins. Based on the literature, the proteins with a confirmed role in neuroprotection occurring in those fractions are murinoglobulin 1 and complement C3.

Besides the common proteins in both samples—characterised by a fixed ratio of fluorescent signals—there was a trend for a reduced area of peak 3 in lane 3 and peak 5 in lane 4 (*p* = 0.058) in the tolerant secretome. Compared with the non-tolerant secretome, there was a 47% reduction for peak 3 of lane 3 and a 48% reduction for peak 5 of lane 4. However, only peak 5 of lane 4 presented a significant reduction in the band percentage, compared with the non-tolerant counterparts (Figure 5). Based on MALDI identification, we classified peak 3 as a mix of proteins listed in Table 1. Based on the literature, kininogen may have the most neuroprotective potential. Peak 5 of lane 4 is apolipoprotein E (ApoE). However, the same protein identified as a peak 5 in lane 3 did not show some changes in peak areas between the tolerant and non-tolerant secretomes (Table 1 and Figure 5).

When examining the lane 3 profile, we found unique bands in the tolerant samples corresponding to peak 2 (Table 1). The average area of the peak was 802.17 ± 142 mm^2^, which corresponded to approximately 10.74 ± 1.9% of the total proteins in lane 3. We also observed this peak in lane 4 (average area of the peak 493.2 ± 284 mm^2^) but not in all analysed samples (Figure 5). Because of the high variability within the group, there were non-significant changes in band percentages, compared with the non-treated counterparts. The variability may have resulted from shifts during excision of individual native gel fractions during processing. MALDI analysis identified a single upregulated protein in the tolerant secretome, namely haptoglobin.

### 3.3. RIC Alters the Functional Interaction of Bioactive Proteins

**Figure 6:**
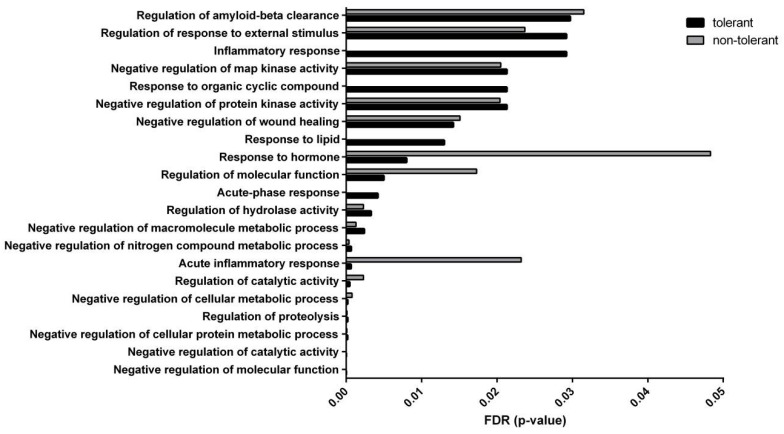
Comparison of the false discovery rate of the significantly enriched gene ontology terms in the bioactive fractions of blood cell-derived secretomes drawn from remote ischaemic conditioning (RIC)-treated and non-treated rats.

We analysed the functional interaction of proteins in the tolerant secretome by the in silico STRING tool. When omitting the downregulated protein peaks (but still presented in the samples based on the lane profile), the only unique protein that appears in the bioactive compound of tolerant secretome is haptoglobin. The proteins in the bioactive fraction of the non-tolerant secretome showed significant enrichment of 17 gene ontology (GO) terms of biological processes. The three with the lowest FDR are ‘Negative regulation of molecular function’ (*p* = 0.00000241), ‘Negative regulation of catalytic activity’ (*p* = 0.0000152), and ‘Negative regulation of cellular protein metabolic process’ (*p* = 0.0000911). The inclusion of haptoglobin to predict protein–protein interactions (which is characteristic of the tolerant secretome) revealed 21 significantly enriched GO terms (Table 2). Of these 21 GO terms, 17 are shared with the non-tolerant secretome and 4 are unique, namely ‘Acute-phase response’ (*p* = 0.0042), ‘Response to lipid’ (*p* = 0.013), ‘Response to organic cyclic compound (*p* = 0.0213), and ‘Inflammatory response’ (*p* = 0.0292). Moreover, the FDR of the GO terms was markedly altered after including haptoglobin (Figure 6). The most notable reduction in the *p*-values was for ‘Negative regulation of cellular metabolic process’, ‘Regulation of catalytic activity’, ‘Acute inflammatory response’, ‘Regulation of molecular function’, and ‘Response to hormone’.

## 4. Discussion

Blood is a circulating fluid consisting of a mutually interacting population of different cells. Red blood cells are the major cell type; they are pleiotropic cells with the capacity to engage in reciprocal cross-talk with nearby cells. In addition to transporting oxygen and carbon dioxide, red blood cells also regulate vascular contractility, T cell growth and survival, and proteinase and interleukin release. It has been reported that red blood cells spontaneously and continuously release vesicles containing erythrocyte proteins and soluble proteins with bioactive effects. Those factors help sustain the maturation and survival of T cells or increase the interaction of platelets with neutrophils and monocytes [11]. The fact that blood cells release proteinaceous substances with bioactive and cytoprotective effects has been widely documented in native conditions, as well as after stimulation. In addition to erythrocytes, differentiated peripheral mononuclear cells seem to have versatile cytoprotective and regenerative properties comparable to stem cells, which are difficult to obtain. The secretome of apoptotic mononuclear cells has been shown to positively influence wound healing [12] and regenerate the myocardium [13] and the brain after stroke [14]. Detailed analysis of the apoptotic mononuclear secretome has shown stimulus-induced changes in the expression of genes that encode secreted proteins related to pro-angiogenic and regenerative pathways. However, the mononuclear secretome stimulates fibroblast gene expression regardless of the stimulus [13]. Similarly, the results of our in vitro studies confirm the neuroprotective effect of the non-stimulated blood cell secretome, although it is less intense, compared with the RIC-treated counterpart. On the other hand, the RIC effect is mediated by the complex response of protein synthesis machinery in terms of conservation, suppression, and stimulation of protein appearance that mediates improved neuronal survival, cell integrity, and metabolic activity after exposure to a toxic dose of glutamate. RIC maintains a constant level of most of the major protein peaks; however, a unique peak appeared and a few were downregulated (but not completely suppressed). Our results are supported by previous findings [15]. The authors observed fluctuation in plasma peptide number and significant up-/downregulation of peptides during the hind limb ischaemia cycles and reperfusion periods in healthy individuals. Surprisingly, the total number of plasma proteins increased during the three RIC cycles and the majority of analysed peptides were upregulated, suggesting a rapid and cumulative response to RIC stimulus [15]. On the contrary, as the post-RIC time increased, the number of plasma proteins and the percentage of significantly downregulated peptides decreased, findings that represent the global proteomic response during the reperfusion period.

In our study, haptoglobin was the only plasma protein significantly elevated in the tolerant secretome; it was an integral part of peak 2 of lane 3 of the tolerant secretome. Haptoglobin is an acute-phase α_2_-acid response glycoprotein. Most of the current information on the structure and function of haptoglobin has been derived from the plasma form and primarily concerns its role as a haemoglobin-binding protein [16] or antioxidant [17]. Extrahepatic haptoglobin expression has been found in some blood-cell types, such as neutrophils [18], dendritic cells, macrophages [19], and B cells [20]. Despite the differences in post-translational modifications of extrahepatic haptoglobin [18], all forms bind haemoglobin and function as antioxidants at the site of neutrophil activation [21]. A recent study demonstrated that haptoglobin administration during post-ischaemic recovery significantly improved functional outcomes, including survival, motor function, and brain damage, by binding to haemoglobin 1 and modulating the polarisation of macrophages/microglia [22]. These findings suggest the potential of haptoglobin as a novel neuroprotective, therapeutic target for brain ischaemia-reperfusion injury. Based on our in silico analysis of the identified proteins in the bioactive fractions of the tolerant secretome, haptoglobin inclusion enhances the biological processes of negative regulation of molecular function and catalytic activity but positively influences regulation of cellular protein metabolic process. At least five proteins recognised as downregulated based on the lane profile and subsequent MS/MS identification are an integral part of those pathways. Moreover, biological processes related to response to acute phase and acute inflammation and answer to inflammation, hormones, and lipids were positively enhanced by haptoglobin.

Numerous studies have shown that RIC regulates several pathways, including the immune system, mitochondrial function, and oxidative stress and angiogenesis (summarised in [2]). The protein profiling of the bioactive fractions of the secretomes uncovered the presence of ceruloplasmin, an antioxidant present in the blood. Although ceruloplasmin was not upregulated in response to RIC in our experiments, recent findings support the role of this protein in ameliorating stroke outcomes. Although the liver provides an important source of serum ceruloplasmin, activated monocytes and macrophages could produce a soluble form of this antioxidant [23] and could be the source of ceruloplasmin in the blood cell-derived secretome. Ceruloplasmin is involved in the conversion of ferrous iron (Fe^2+^, widely produced during post-ischaemic reperfusion) to less toxic ferric iron (Fe^3+^) [24]. In addition, the ferroxidase activity of ceruloplasmin inhibits Fe^2+^-mediated production of reactive oxygen species; thus, ceruloplasmin possesses a potent antioxidant activity [25].

The neuroprotective effect of RIC treatment is mediated by immunomodulation in stroke patients. We identified two potential immunomodulatory proteins in the bioactive fractions of the blood cell-derived secretome, namely murinoglobulin 1 and complement C3, although the relative concentrations were stable. Murinoglobulin 1 has been established as an important immunomodulatory protein produced by macrophages in myocardial infarction. Although neutrophil degranulation is essential to create an optimum environment for later scar formation, moderation is needed to prevent excessive infarct wall thinning. Murinoglobulin 1 secreted by macrophages in a mouse model inhibited neutrophil degranulation on the side of injury and pose as an endogenous mechanism to temper neutrophil degranulation [26]. Although RIC modulates innate and adaptive circulating immune cells during the first 24 h [27], we have not found some information that the direct monocyte ischaemia/hypoxia or RIC could activate monocyte to macrophage transformation and MUG1 release.

The complement system is an important part of the innate immune defence. Complement C3 expression in human serum significantly decreases from 1 to 24 h after RIC, a phenomenon that is believed to play a significant role in RIC-mediated neuroprotection [28]. Those observations are supported by findings that only C3-deficient mice show significant neuroprotection when subjected to cerebral infarction [29]. Most of the complement proteins are synthesised by hepatocytes [30]. However, there is a growing body of evidence that local secretion of complement proteins plays an important role in regulating physiological processes. Local secretion by blood cells could explain the constant level of C3 in plasma-free samples of blood cells cultivated ex vivo instead of C3 plasma reduction in RIC-treated individuals. Immune system cells can be included in complement production (reviewed in [31]). For example, activated polymorphonuclear leucocytes secrete complement C3 [32], and its production can also be stimulated by irradiation [13] and by RIC in blood cells [6]. Taken together, complement C3 has been implicated as a potential marker of stimulation-mediated secretion of blood cells.

When focussing on the proteins related to the downregulated peaks in the bioactive fractions of the tolerant secretome, kininogen, and ApoE could represent potential sources of cytoprotection. Kininogen 1 is an important constituent of the plasma contact–kinin system, a network of serially connected serine proteases [33]. Activation of the contact–kinin system triggers cleavage of kininogen by plasma kallikrein and subsequent release of the proinflammatory peptide hormone bradykinin. In acute ischaemic stroke, activation of the contact–kinin system fosters vascular permeability and stroke-related inflammation by the formation of short-lived kinins [34]. On the other hand, bradykinin has been used as a cytoprotective agent against the heart [35], as well as the brain and spinal cord ischaemia [36,37,38]. It is believed that the neuroprotective effect of bradykinin is related to the stress reaction of the organism; its application increases tolerance to ischaemic conditions (pre/postconditioning), so-called ischaemic tolerance. Considering our results, we speculate that kininogen cleavage to the low-molecular-weight peptide bradykinin could explain the drop in the protein content of peak 3 after RIC. Subsequently, bradykinin resulting from this cleavage acts as a tolerance-inducing stimulus and increases neuronal sensitivity to a toxic dose of glutamate. However, due to the low molecular weight of bradykinin and inappropriate sensitivity of the MS/MS instrumentation used for protein profiling in our experiments, this hypothesis should be tested with another set of experiments.

ApoE plays a pivotal role in both peripheral and cerebral cholesterol metabolism. In plasma, ApoE is mainly carried by triglyceride-rich lipoproteins and serves as a ligand for members of the low-density lipoprotein receptor family [39]. Low plasma ApoE levels are associated with an increased risk of dementia [40], whereas high levels are associated with an increased risk of ischaemic heart disease and diabetes [41]. In the brain, ApoE is believed to play a key function in the clearance of ischaemia-damaged brain tissue and tissue remodelling. ApoE is synthesised and released by astrocytes after injury and may be taken up by degenerating neurons. After infarction, macrophages continue to accumulate in the infarct tissue for about 1 week [42] and are induced to express ApoE [43]. There is currently no clear evidence about the influence of RIC on ApoE; however, other classes of apolipoproteins, such as ApoD [44] and ApoA1 [28], are significantly reduced immediately after RIC. On the other hand, there was an increased plasma level of ApoA1 in RIC-treated rats 5 and 10 min after the last limb ischaemia cycle, suggesting the time dependence of plasma apolipoprotein levels on poststimulation recovery.

## 5. Conclusions

We conclude that blood cells can release bioactive proteins/protein complexes in native conditions, but RIC markedly improves their cytoprotective potency. The effect of RIC stimulation promotes a balance between conservation, suppression, and stimulation of blood cell-released proteins. We identified 18 proteins in the bioactive fractions of the secretomes; however, only 1, haptoglobin, was uniquely secreted in the tolerant secretome. Haptoglobin secretion enhances negative regulation of molecular function and catalytic activity; positively impacts the regulation of cellular protein metabolic process; and increases biological processes related to acute phase and inflammation and the response to inflammation, hormones, and lipids. Although we have narrowed down the proteins of blood cell-derived secretomes to those that exhibit the greatest cytoprotective effects, additional studies are needed to elucidate their particular function and detailed mechanisms of action.

## Figures and Tables

**Table 1:** The proteins identified in biologically active fractions of blood cell-derived secretomes drawn from remote ischaemic conditioning (RIC)-stimulated (tolerant) and non-RIC-stimulated (non-tolerant) rats. The biologically active content of the blood secretome was divided into fractions according to the distance from the top of the separation gel after electrophoretic separation and assigned to peaks according to the lane. The peak area (mm^2^) and the percentage of the total area of the peaks in lane (band %) and *p*-value, resulting from statistical comparisons of the datasets are presented. (* *p* < 0.05).

Distance (mm)	Peak Sequence	Area (mm^2^)		Area (*p*-Value)	Band (%)		Band% (*p*-Value)	Protein
		Non-Tolerant (CH1)	Tolerant (CH2)		Non-Tolerant	Tolerant		
**Line 3**								
5	peak 1	2277.53 ± 144	1669.68 ± 205	0.0540651	39.99 ± 6	36.57 ± 5.7	0.685846	Murinoglobulin-2
10								Alpha-1-inhibitor 3
								Complement C3
15								Complement C3
								Guanine nucleotide-binding protein subunit alpha-13
20	peak 2	X	802.17 ± 142	0.00135494 *	X	10.74 ± 1.9	0.000471288 *	Haptoglobin
25	peak 3	1162.79 ± 99	789.13 ± 70	0.0211761 *	12.34 ± 1.2	15.68 ± 1.8	0.163233	Centrosomal protein of 70 kDa
								Glycerol-3-phosphate dehydrogenase, mitochondrial
								3-hydroxy-3-methylglutaryl-coenzyme A reductase
								Kininogen-1 OS = Rattus norvegicus
								Thyroid hormone receptor alpha
30	peak 4	675.5 ± 43	696.3 ± 129	0.882889	7.11 ± 1.1	8.47 ± 2.7	0.64914	Fetuin-B OS = Rattus norvegicus
35								
40	peak 5	685.5 ± 260	880.08 ± 185	0.564531	5.06 ± 3.1	3.34 ± 1.5	0.628697	Apolipoprotein E
45	peak 6	1620.67 ± 142	1650.91 ± 144	0.889648	35.4 ± 5.8	24.16 ± 5.8	0.206228	Collagen alpha-2(I) chain
								POU domain, class 4, transcription factor 2
**Line 4**								
5	peak1	1814.12 ± 206	1368.27 ± 181	0.141957	11.88 ± 3.1	21.03 ± 4.1	0.102411	
10								Murinoglobulin-1
15								Ceruloplasmin
20	peak 2	X	493.21 ± 284	0.0885759	X	4.32 ± 1.5	0.0652243	Haptoglobin
25	peak 3	381.37 ± 165	635.92 ± 165	0.307385	2.18 ± 1.2	5.12 ± 2	0.237078	Alpha-2-HS-glycoprotein
30	peak 4	1015.21 ± 111	840.07 ± 156	0.387032	8.92 ± 1.2	13.94 ± 3.5	0.208264	Alpha-2-HS-glycoprotein
35								
40	peak 5	1529.37 ± 171	1030.58 ± 59	0.058	31.3 ± 4.3	8.97 ± 1.4	0.000596955 *	Apolipoprotein E
45	peak 6	1559.96 ± 318	1432.38 ± 161	0.7299	45.61 ± 6.7	44.45 ± 4.8	0.891795	Protein polyglycylase TTLL10

**Table 2:** List of significantly enriched biological processes in the biologically active fraction of the blood cell-derived secretome drawn from rats subjected to remote ischaemic conditioning.

#Term ID	Term Description	Matching Proteins
GO:0044092	Negative regulation of molecular function	Thra, Pou4f2, Mug1, Hmgcr, A1i3, Hp, Apoe, Fetub, Ahsg, Mug2, Kng2, C3
GO:0043086	Negative regulation of catalytic activity	Mug1, Hmgcr, A1i3, Hp, Apoe, Fetub, Ahsg, Mug2, Kng2, C3
GO:0032269	Negative regulation of cellular protein metabolic process	Mug1, Hmgcr, A1i3, Apoe, Fetub, Ahsg, Mug2, Kng2, C3
GO:0030162	Regulation of proteolysis	Mug1, A1i3, Apoe, Fetub, Ahsg, Mug2, Kng2, C3
GO:0031324	Negative regulation of cellular metabolic process	Thra, Pou4f2, Mug1, Hmgcr, A1i3, Hp, Apoe, Fetub, Ahsg, Mug2, Kng2, C3
GO:0050790	Regulation of catalytic activity	Mug1, Hmgcr, A1i3, Hp, Apoe, Fetub, Gna13, Ahsg, Mug2, Kng2, C3
GO:0002526	Acute inflammatory response	Mug1, Hp, Ahsg, Kng2
GO:0051172	Negative regulation of nitrogen compound metabolic process	Thra, Pou4f2, Mug1, Hmgcr, A1i3, Apoe, Fetub, Ahsg, Mug2, Kng2, C3
GO:0010605	Negative regulation of macromolecule metabolic process	Thra, Pou4f2, Mug1, Hmgcr, A1i3, Apoe, Fetub, Ahsg, Mug2, Kng2, C3
GO:0051336	Regulation of hydrolase activity	Mug1, A1i3, Fetub, Gna13, Ahsg, Mug2, Kng2, C3
GO:0006953	Acute-phase response	Mug1, Hp, Ahsg
GO:0065009	Regulation of molecular function	Thra, Pou4f2, Mug1, Hmgcr, A1i3, Hp, Apoe, Fetub, Gna13, Ahsg, Mug2, Kng2, C3
GO:0009725	Response to hormone	Thra, Pou4f2, Col1a2, Hp, Apoe, Ahsg, C3
GO:0033993	Response to lipid	Thra, Pou4f2, Col1a2, Hmgcr, Hp, Apoe, C3
GO:0061045	Negative regulation of wound healing	Hmgcr, Apoe, Kng2
GO:0006469	Negative regulation of protein kinase activity	Hmgcr, Apoe, Ahsg, Kng2
GO:0014070	Response to organic cyclic compound	Thra, Pou4f2, Col1a2, Hmgcr, Hp, Apoe, C3
GO:0043407	Negative regulation of map kinase activity	Hmgcr, Apoe, Kng2
GO:0006954	Inflammatory response	Mug1, Hp, Ahsg, Kng2, C3
GO:0032101	Regulation of response to external stimulus	Pou4f2, Hmgcr, Apoe, Ahsg, Kng2, C3
GO:1900221	Regulation of amyloid-beta clearance	Hmgcr, Apoe

## Data Availability

Not applicable.

## Supplementary Materials

The following supporting information can be downloaded at: https://www.mdpi.com/article/10.3390/biom12101423/s1, Figure S1: Detailed workflow of the experiment.
