# Supplementary material for: Identification of Proteins Responsible for the Neuroprotective Effect of the Secretome Derived from Blood Cells of Remote Ischaemic Conditioned Rats
Source: Biomolecules. 2022 Oct 4;12(10):1423. doi: 10.3390/biom12101423 (PMC9599669; doi:10.3390/biom12101423)
Supplement: Supplementary file 1 [file biomolecules-12-01423-s001.zip › biomolecules-1910018-supplementary.pdf]

Supplementary information for:

Title: **Identification of Proteins Responsible for the Neuroprotective Effect of the Secretome Derived From Blood Cells of Remote Ischaemic Conditioned Rats**

Petra Bonova [1,*], Jana Koncekova [1], Miroslava Nemethova [1], Klaudia Petrova [1], Martin Bona [2] and Miroslav Gottlieb [1]

[1] Institute of Neurobiology, Biomedical Research Center of the Slovak Academy of Sciences, Soltesovej 4-6, 040 01 Košice, Slovakia
[2] Department of Medical Physiology, Faculty of medicine, University of Pavol Jozef Safarik, 040 01 Košice, Slovakia
* Correspondence: bonova@saske.sk

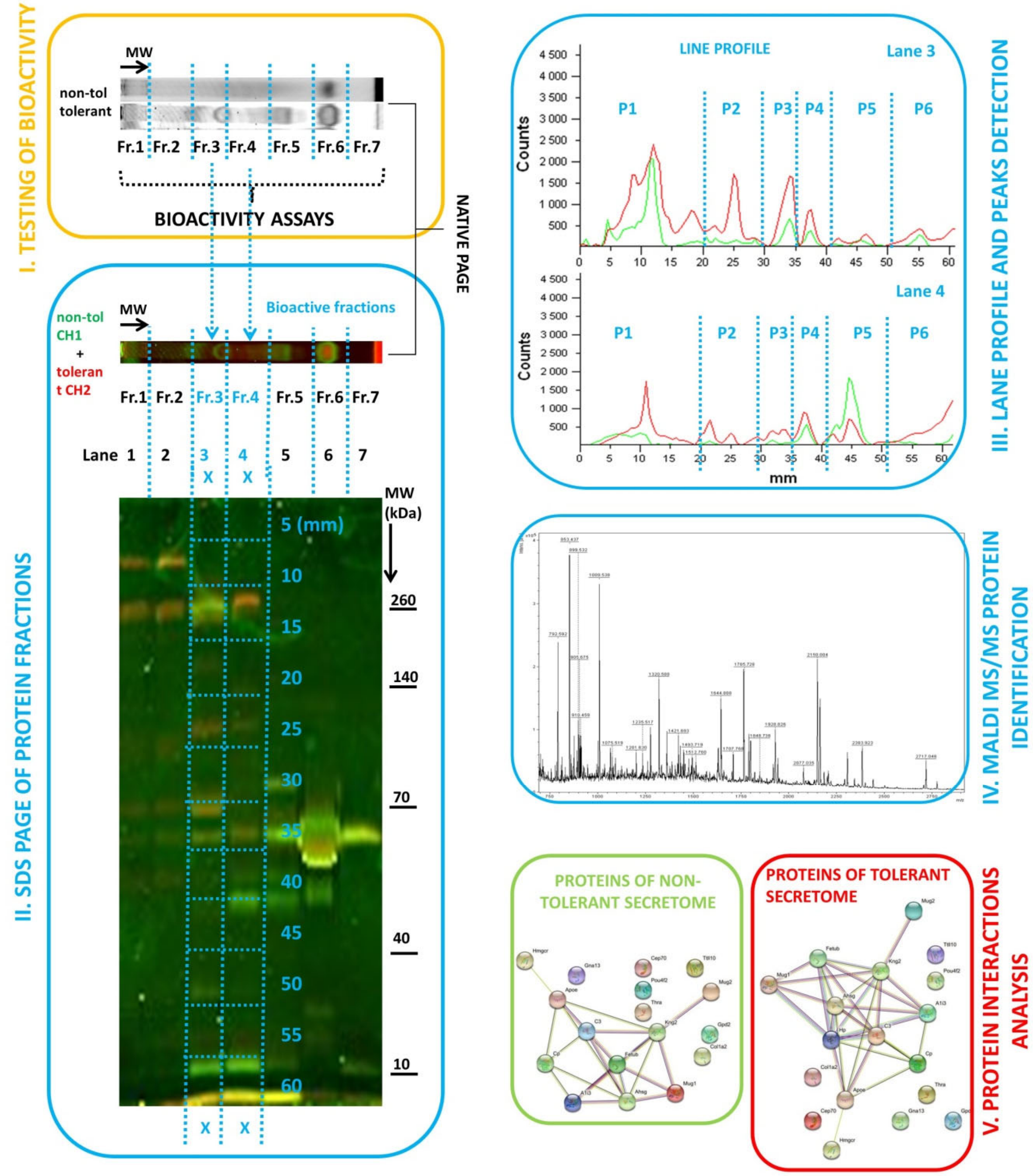

Figure S1. Detailed workflow of the experiment.

The blood cells of RIC-treated and non-treated animals were incubated *ex vivo* to compare protein changes triggered by RIC. The proteins of tolerant and non-tolerant secretomes were labelled with two variants of Cy fluorescent dyes (Cy3 and Cy5) – each with different excitation and emission spectra – then the samples were mixed and separated into seven fractions with native PAGE. After the denaturation and separation of the proteins with SDS-PAGE and the profiles of each lane was examined. Based on the density of the bands, the peak area was calculated to identify unique protein bands/peaks and to calculate semi-quantitatively changes in common peaks. The main goal was to document the profiles of lanes 3 and 4 (fractions 3 and 4, respectively) and profiled lanes 3 and 4 with MALDI TOF/TOF. Nine pieces (0.5 cm) were excised from each lane and individually analysed to identify proteins. Based on the peak layouts, the lane profile was divided into six parts. The peak area (mm^2) and its percentage of the total area of the peaks in the lane (band %) were calculated, and the locations on the lane profile was defined (mm from the top of the lane).
